# White matter microstructure, traumatic brain injury, and disruptive behavior disorders in girls and boys

**DOI:** 10.3389/fnins.2024.1391407

**Published:** 2024-07-19

**Authors:** Guido I. Guberman, Guillaume Theaud, Samuel W. Hawes, Alain Ptito, Maxime Descoteaux, Sheilagh Hodgins

**Affiliations:** ^1^Department of Neurology and Neurosurgery, Faculty of Medicine, McGill University, Montreal, QC, Canada; ^2^Department of Computer Science, Sherbrooke University, Sherbrooke, QC, Canada; ^3^Department of Psychology, Center for Children and Families, Florida International University, Miami, FL, United States; ^4^Département de Psychiatrie et Addictologie, Université de Montréal, Montreal, QC, Canada; ^5^Centre de Recherche Institut National de Psychiatrie Légale Philippe-Pinel, Montreal, QC, Canada

**Keywords:** traumatic brain injury, diffusion MRI (dMRI), tractometry, behavior problems, multivariate analysis

## Abstract

**Introduction:**

Girls and boys presenting disruptive behavior disorders (DBDs) display differences in white matter microstructure (WMM) relative to typically developing (TD) sex-matched peers. Boys with DBDs are at increased risk for traumatic brain injuries (TBIs), which are also known to impact WMM. This study aimed to disentangle associations of WMM with DBDs and TBIs.

**Methods:**

The sample included 673 children with DBDs and 836 TD children, aged 9–10, from the Adolescent Brain Cognitive Development Study. Thirteen white matter bundles previously associated with DBDs were the focus of study. Analyses were undertaken separately by sex, adjusting for callous-unemotional traits (CU), attention-deficit hyperactivity disorder (ADHD), age, pubertal stage, IQ, ethnicity, and family income.

**Results:**

Among children without TBIs, those with DBDs showed sex-specific differences in WMM of several tracts relative to TD. Most differences were associated with ADHD, CU, or both. Greater proportions of girls and boys with DBDs than sex-matched TD children had sustained TBIs. Among girls and boys with DBDs, those who had sustained TBIs compared to those not injured, displayed WMM alterations that were robust to adjustment for all covariates. Across most DBD/TD comparisons, axonal density scores were higher among children presenting DBDs.

**Discussion:**

In conclusion, in this community sample of children, those with DBDs were more likely to have sustained TBIs that were associated with additional, sex-specific, alterations of WMM. These additional alterations further compromise the future development of children with DBDs.

## 1 Introduction

Disruptive behavior disorders (DBDs) that include mainly conduct disorder and oppositional defiant disorder, are diagnosed in children who display persistent rule breaking and aggressive behavior, who constitute the primary risk factor for adult antisocial behavior and criminality (Rivenbark et al., 2018). DBDs are estimated to affect 3% to 12% of children, more boys than girls (Nock et al., 2007). The population presenting DBDs is heterogeneous with respect to callous-unemotional traits (CU) presented by ~40% of girls and boys (Nock et al., 2007), and Attention Deficit Hyperactivity Disorder (ADHD) presented by 24% of the boys and 36% of the girls (Waschbusch, 2002). CU is associated with more severe conduct problems, aggressive behavior, and outcomes (Frick et al., 2014).

DBDs are disorders of atypical development of gray and white matter (Blair et al., 2014). Two meta-analyses of magnetic resonance imaging (MRI) studies of DBDs reported that most alterations were observed in gray matter structures of the limbic region (Aoki et al., 2014; Rogers and De Brito, 2016). A meta-analysis of fMRI studies, showed deficits in emotion processing, during tasks of both hot and cool executive functions (Alegria et al., 2016). A recent review concluded that alterations of white matter microstructure (WMM) were associated with antisocial behavior, noting however that results in children were inconclusive as to the implicated tracts and the direction of effects (Waller et al., 2017). Among children with DBDs, the most frequently reported alterations of WMM are found in the uncinate fasciculus (UF) (Waller et al., 2017; Bolhuis et al., 2019) and the corpus callosum (CC) (Puzzo et al., 2018; Rogers et al., 2019), consistent with findings among adults with Antisocial Personality Disorder who by definition presented DBDs in childhood (Association, 2013). One study of teenage boys and girls with CD reported lower FA and hindrance-modulated orientational anisotropy in the right retrosplenial cingulum relative to typically developing (TD) peers with sex differences such that males with CD displayed significantly lower FA compared to TD males while females with and without CD did not differ (González-Madruga et al., 2020).

The combination of DBDs and CU has been associated with differences in the UF (Puzzo et al., 2018; Bolhuis et al., 2019) and the CC (Rogers et al., 2019) among girls with conduct disorder (Menks et al., 2017). When controlling for CU, the same bundles (tracts) have been found to be associated with DBDs (Rogers et al., 2019). A study of a large birth cohort from The Netherlands reported that CU, not accounting for DBDs, was associated with differences in global mean diffusivity driven by the UF, cingulum and CC in girls not boys (Bolhuis et al., 2019).

The few studies of the association of DBDs and WMM focused almost exclusively on teenage boys, and did not account for several factors known to affect WMM such as maltreatment (Jaffee et al., 2005) and substance misuse (Armstrong and Costello, 2002) that are more common among children with DBDs than those who are typically developing (TD). Further, almost all studies employed the diffusion tensor model, which, in the presence of underlying fiber heterogeneity, cannot disentangle the contributions of different neuropathologies to WMM. Thus, the extant literature on WMM associated with DBDs is limited and inconsistent, but suggestive of alterations of tracts connecting limbic regions, and sex-specific differences.

Importantly, previous studies of children with DBDs did not take account of traumatic brain injuries (TBIs). Yet, boys with DBDs are at increased risk of accidents (Rivara, 1995) and of TBIs (Guberman et al., 2020b) as compared to their peers. In a sample of 628 males, age 10 teacher ratings of inattention-hyperactivity predicted TBIs up to age 34, and ratings of a composite score for externalizing problems predicted TBIs from age 18 to 34, after accounting for previous TBIs and family social status (Guberman et al., 2020b). Decades of research have shown that TBIs impact WMM in children (Hulkower et al., 2013; Dodd et al., 2014). Could TBIs play a role in altering WMM of children with DBDs thereby adding to, or compounding, the existing atypical neurodevelopment?

There are few studies of WMM in children who have sustained TBIs (Dodd et al., 2014) and none to our knowledge that accounted for DBDs. In small samples of children/adolescents who had sustained TBIs, alterations have been observed in WMM maturation rates (Ewing-Cobbs et al., 2008) including in the CC (Beauchamp et al., 2011). Few studies have investigated sex differences in WMM alterations following a concussion. One study of 244 children and adolescents who had sustained mild-to-severe TBIs and 263 matched non-injured peers reported that girls, not boys, exhibited lower fractional anisotropy in the UF (Dennis et al., 2021).

Children with DBDs present high risks for antisocial and/or criminal behavior in adolescence and adulthood, and evidence of deficits in emotion processing (Sully et al., 2015) and executive functions (Hobson et al., 2011) from childhood onwards. The key tracts subserving these functions have been reported to be altered relative to TD individuals, in a sex-specific manner. In the same sample as studied in the present report, WMM across the brain was inversely associated with executive functions, and via executive functions it was indirectly associated with conduct problems and ADHD (Cardenas-Iniguez et al., 2022). It is therefore possible that WMM differences reported in comparisons between DBD and TD youth may be attributable to a greater incidence of TBIs in children with DBDs. It is also possible that differences reported in comparisons between children with TBIs and those without may be confounded by the more likely presence of DBDs in the injured children. Our study aimed to tease apart the influence of these two conditions on WMM.

### 1.1 The present study

We used data collected on 11,875 children aged 9 to 10 years old from the Adolescent Brain Cognitive Development (ABCD) Study (Casey et al., 2018). We aimed to determine, for the first time, whether children with DBDs who had not experienced a TBI display differences in WMM relative to non-injured TD children. Next, we examined children who had sustained TBIs, and determined whether those with DBDs, relative to TD children, showed alterations in WMM. Finally, we determined whether TBIs were associated with the same differences in WMM among children with DBDs and among TD children. We expected that given the distinctive neural development characterizing DBD children, TBIs would be associated with alterations to structures distinct from those in the TD children.

Girls, as compared to boys, are less likely to present DBDs, obtain lower scores for CU (Frick et al., 2014), display faster white matter development (Schmied et al., 2020), mature more quickly, and have a higher risk of TBIs and of serious negative outcomes (Baker et al., 2016). Among children with DBDs, much less is known about girls than boys, including differences in WMM as compared to healthy peers. Therefore, we examined boys and girls separately, hoping to maximize knowledge gained from this rare large sample of girls presenting DBDs. Within each sex, we controlled for pubertal stage and age.

Since previous studies suggested that among children with DBDs, CU was associated with differences in WMM, perhaps in a sex-specific manner, analyses controlled for CU. Because comorbid inattention-hyperactivity has been found to be associated with distinct alterations of WMM, analyses also controlled for Attention Deficit Hyperactivity Disorder (ADHD).

In addressing these questions, the present study employed novel modeling and tractography techniques that are robust to the biases of diffusion tensor imaging (DTI) and novel statistical approaches (described in Supplementary material) that leverage the information contained within diffusion measures to more exhaustively probe WMM.

## 2 Methods and materials

### 2.1 Participants

Data were obtained from the ABCD study (https://abcdstudy.org/) 2.0 data release (https://data-archive.nimh.nih.gov/abcd) that recruited 11,875 healthy children, aged 9 to 10 years from across the United States (48% girls). The sample, procedures, and ethics approval have been described previously (Garavan et al., 2018), and exclusion criteria can be found in Karcher et al. (2018).

### 2.2 Measures

#### 2.2.1 Disruptive behavior disorders

As previously described (Waller et al., 2020), children's behaviors were rated by one parent using self-administered computerized versions of the Child Behavior Checklist (CBCL) and the Schedule for Affective Disorders and Schizophrenia for school-age children (KSADS). A KSADS past or current diagnosis of conduct disorder and/or oppositional defiant disorder and/or CBCL scores on these scales of 66 or higher were coded as DBDs. Psychometric properties of this measure are described elsewhere and are considered to be strong (Waller et al., 2020).

#### 2.2.2 CU traits

We quantified CU traits using a measure derived and validated on ABCD Study participants and an independent sample of children that showed good psychometric properties, measurement invariance across sex, race, and age, and differences from conduct problems, oppositional defiant disorder, and ADHD, and meaningful associations with outcomes (Hawes et al., 2020). The measure of CU includes one item from the parent-rated CBCL (“lack of guilt after misbehaving”) and three items from the parent rated Strengths and Difficulties Questionnaire (“is considerate of others' feelings”; “is helpful if someone is hurt or upset”; “offers to help others”) (all three reverse-coded) rated on a three-point scale (0–1–2). Following the procedures described in two prior studies (Hawes et al., 2020; Waller et al., 2020), we obtained two CU measures from these items, the sum of responses, and a *maximum a posteriori* (MAP) scores that accounts for which items are endorsed by whom, providing person-specific factor scores for CU traits. We then dichotomized these scores defining the presence of high CU traits as a summed score of 4 or above and a CU traits MAP score at or above the 90th percentile.

#### 2.2.3 Attention-deficit hyperactivity disorder

Attention-deficit hyperactivity disorder was indexed using T-scores from the DSM-oriented Attention Problems scale from the CBCL.

#### 2.2.4 Pubertal stage

Parents completed the Pubertal Development Scale and Menstrual Cycle Survey History questionnaire (Cheng et al., 2021). Responses were tallied by the ABCD Study team into three stages of pubertal development.

#### 2.2.5 Intelligence Quotient (IQ)

Children completed a computerized version of the National Institutes of Health Toolbox Cognitive function Battery (Akshoomoff et al., 2013) that assessed seven cognitive abilities. The ABCD Study team computed fully corrected T-Scores, comparing the score of the participant to those in the NIH Toolbox nationally representative normative sample, adjusted for age, gender, race/ethnicity, and parental educational attainment. We used this fully-corrected score as a measure of IQ.

#### 2.2.6 Traumatic brain injuries

Parents completed a modified version of the Ohio State University TBI Identification Method (Corrigan and Bogner, 2007). We defined a mild TBI as a head injury without loss of consciousness but with memory loss and/or a head injury with loss of consciousness for < 30 minutes. In the present study, all TBIs met this definition of mild TBI. Although history of a severe TBI was part of the exclusion criteria (Karcher et al., 2018), in the whole ABCD sample (*n* = 11,875), seven children had sustained TBIs of moderate or high severity. None of them met criteria for either the DBD or the TD group.

#### 2.2.7 Sociodemographic measures

Parents completed the Parent Demographics Survey to report on their child's ethnicity, family income, their own education, and marital status.

#### 2.2.8 Parent substance use

Parents completed the Family History Assessment to report on substance use.

### 2.3 Group classification

**Figure 1:**
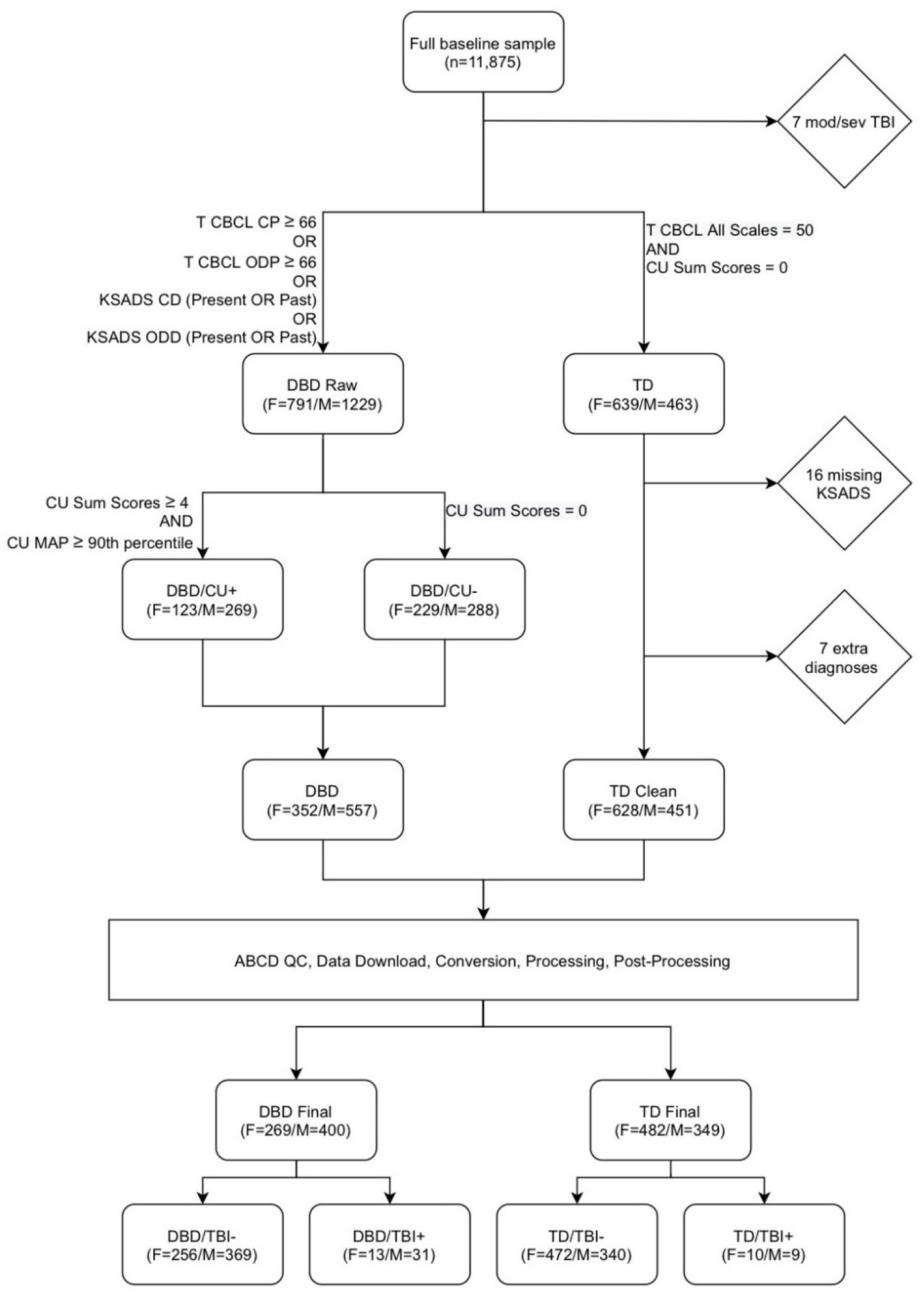
Flowchart describing participant selection. From the full baseline sample (*n* = 11,875), after excluding participants with moderate or severe TBI (“mod/sev TBI”), a disruptive behavior disorders (DBD raw) and a typically developing (TD) group were created. The DBD raw group was defined as Child Behavior Checklist (CBCL) T-scores of 66 or higher on either the conduct problems (CP) or oppositional defiant problems (ODP) scales or a diagnosis of present or past Conduct Disorder (CD) or Oppositional Defiant Disorder (ODD) on the Schedule for Affective Disorders and Schizophrenia for school-age children (KSADS). The “TD” group was defined as individuals with no DBDs and no callous-unemotional traits (CU), and T scores of 50 on all CBCL scales. From the TD group, we excluded 16 participants who had missing diagnostic data, and 7 who had other diagnoses. We identified participants without CU, defined as CU Sum Scores of 0, and participants with high CU, defined as CU sum scores of 4 or more and a *maximum a posteriori* (MAP) CU scale score scores at or above the 90th percentile. These two groups were combined to create a DBD group with approximately half of the members with high CU, and half with no CU. Scans underwent pre-processing, processing, and post-processing, leading to the exclusion of 240 DBD participants and 248 TD participants due to missing or corrupt data files, poor image quality, and failures during image processing and post-processing. The final groups of DBD and TD participants were then divided according to history of traumatic brain injury (TBI).

Figure 1 presents the selection and exclusion criteria that led to a final sample of 751 girls and 749 boys. From the full baseline sample (*n* = 11,875), within each sex, a DBD (“DBD raw”) and a TD group were created. The DBD group was defined to include children with Child Behavior Checklist T-scores of 66 or higher on either the conduct problems or oppositional defiant problems scales or a diagnosis of present or past Conduct Disorder or Oppositional Defiant Disorder on the KSADS. As previous studies have indicated that WMM differences may be associated with CU rather than directly with DBDs (Puzzo et al., 2018), within this group we included participants with and without CU. We identified participants without CU, defined as CU sum scores of 0, and participants with high CU, defined as CU sum scores of four or more and CU MAP scores at or above the 90th percentile. These two sub-groups were combined to create a DBD group in which approximately half of the members had high CU and half no CU. The TD group was defined as individuals with no DBDs and no CU traits, and T scores of 50 on all Child Behavior Checklist scales. From the TD group, we excluded 16 participants who had missing diagnostic data, and seven who had other diagnoses.

Scans underwent pre-processing, processing, and post-processing, leading to the exclusion of 240 DBD participants and 248 TD participants due to missing or corrupted data files, poor image quality, and failures during image processing and post-processing. The final groups of DBD (boys *n* = 557; girls *n* = 352) and TD (boys *n* = 451; girls *n* = 628) participants were then divided according to presence or absence of at least one TBI.

### 2.4 Magnetic resonance imaging

We used multi-shell diffusion MRI (dMRI) and T1-weighted scans. Only scans rated “high quality” by the ABCD Study team were retained. In pre-processing, we verified that all participants had the necessary image requirements. We processed dMRI and T1-weighted scans using Tractoflow (Theaud et al., 2020), following steps outlined in Theaud et al. (2020). Deviations from the default parameters were the use of white matter seeding and using 12 seeds-per-voxel for tractography.

**Figure 2:**
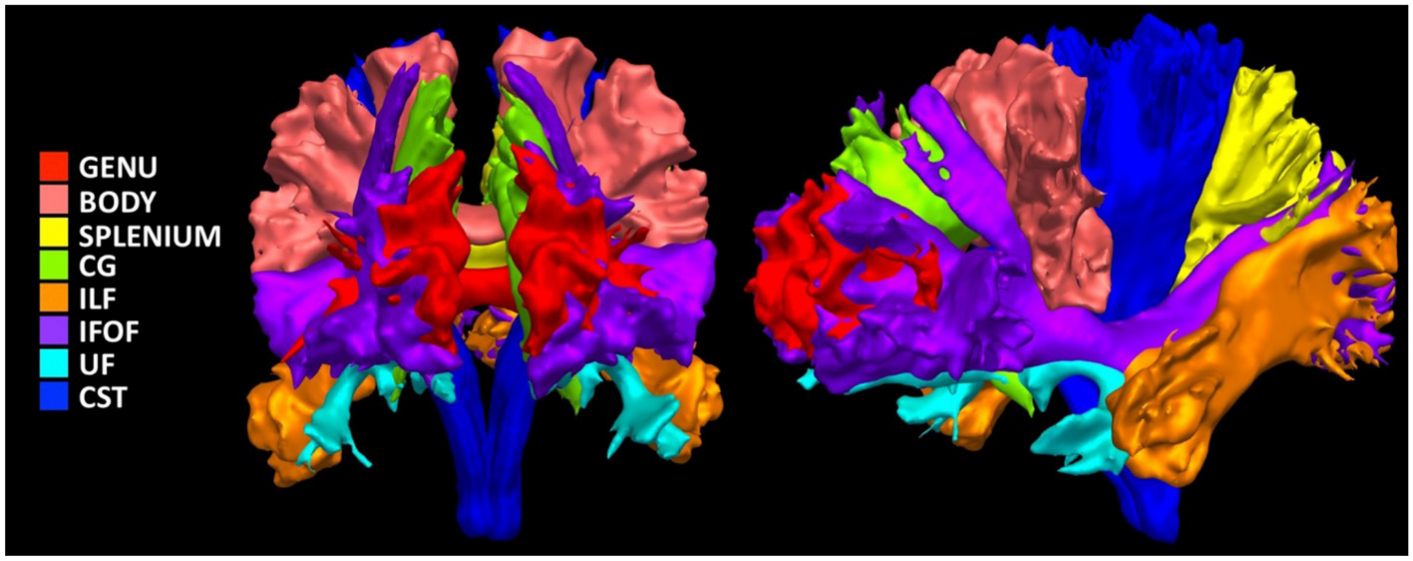
Illustration of the investigated white matter bundles. Genu, Genu of corpus callosum; Body, Body of corpus callosum; Splenium, Splenium of corpus callosum; CG, Cingulum; ILF, Inferior Longitudinal Fasciculus; IFOF, Inferior Fronto-Occipital Fasciculus; UF, Uncinate Fasciculus; CST, Corticospinal Tract.

We used *RBX-flow* (Rheault, 2020) to extract 13 major white matter bundles previously found to differ in children with DBDs (Waller et al., 2017; Puzzo et al., 2018; Bolhuis et al., 2019; Rogers et al., 2019): bilateral UF, inferior fronto-occipital fasciculus (IFOF), cingulum, inferior longitudinal fasciculus (ILF), corticospinal tract (CST), and three portions of the CC, genu, body, and splenium (Figure 2). We then obtained six scalar measures averaged across bundles: fractional anisotropy, mean, radial, and axial diffusivity, number of fiber orientations (Dell'Acqua et al., 2013), and apparent fiber density along fixels (Raffelt et al., 2012).

Partial volume effects, subtle imperfections in brain tissue classification, and other potential errors introduced during tractography can prevent automatic bundling algorithms from extracting bundles (Rheault et al., 2020). Out of 1500 participants with complete data, 386 had at least one bundle that could not be extracted. The lowest number of bundles a participant had was six. Out of all 19500 bundles to extract (1500 participants x 13 bundles/participant), 765 (3.92%) were missing. We imputed missing connectivity data using a non-parametric simple random imputation approach, by randomly selecting data from other participants separately for each bundle and each diffusion measure. We selected this technique because of its simplicity and ease of implementation.

#### 2.4.1 Multidimensional microstructural features

**Figure 3:**
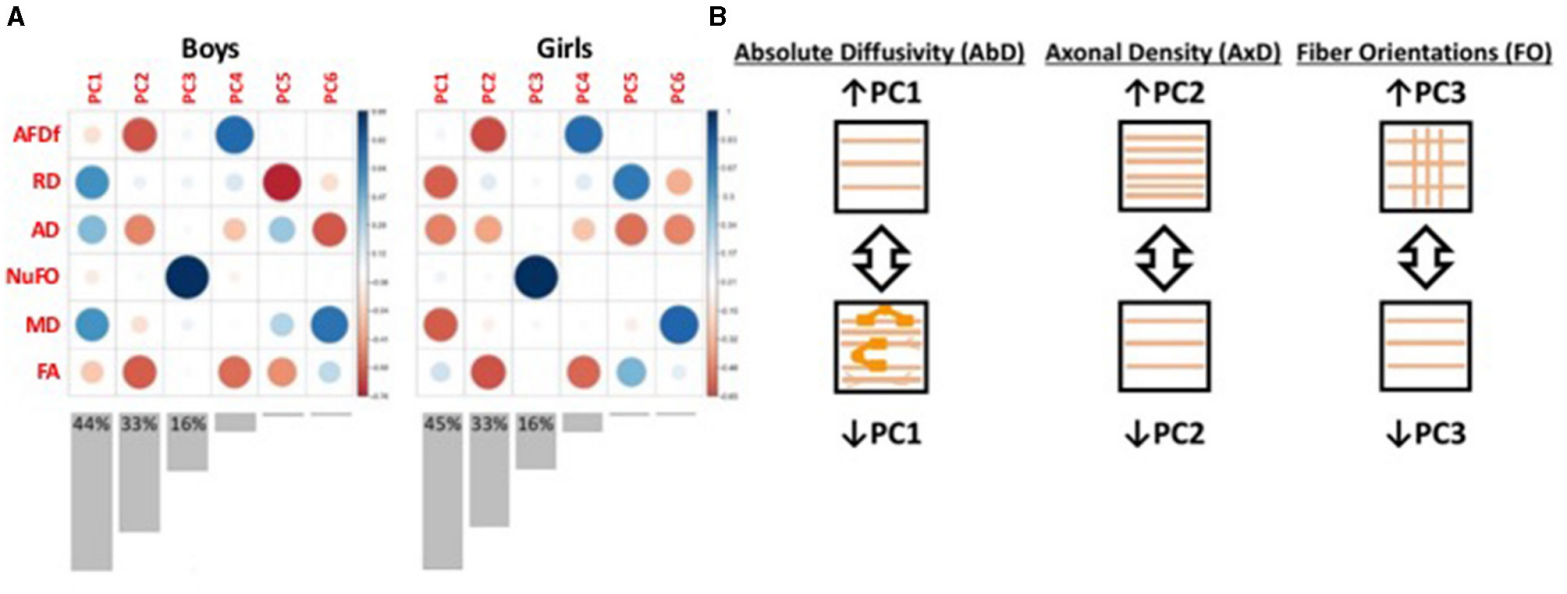
Illustration of the results from the principal components analyses. **(A)** Plot illustrating the loadings of each diffusion measure onto each principal component (PC). Red colors represent negative loadings, blue colors represent positive loadings. The size of the circles also illustrate the magnitude of the loadings. Bar graphs underneath illustrate the variance explained by each PC, with the variance explained by the first three PCs indicated numerically on the graphs. **(B)** Schematic illustration of the interpretation of the first three PCs. PC1 appears to capture measures of absolute diffusivity. The loadings in girls were multiplied by −1 to ensure consistency with the boys' loadings. PC2 appears to capture measures related to axonal density. The loadings in both boys and girls were multiplied by -1 so that increasing PC2 scores could be interpreted as increasing axonal density. PC3 appears to capture selectively the number of fiber orientations. AFDf, Apparent Fiber Density along fixels; RD, Radial diffusivity; AD, Axial diffusivity; NuFO, Number of fiber orientations; MD, Mean diffusivity; FA, Fractional Anisotropy.

Measures from dMRI provide partly overlapping information about underlying microstructure. Used in combination, these measures can provide more information than in parallel (Guberman et al., 2020a, 2022). To extract this shared information, we used principal component analysis (PCA) on the concatenated set of standardized measures across subjects and bundles (Supplementary Figure S1). This approach generated new biologically-interpretable indices of WMM (Chamberland et al., 2019). We applied this technique on the TD group, separately for boys and girls, to obtain measures representative of neurotypical WMM. In both sexes, we obtained three principal components (PCs) that together accounted for 93%−94% of the total variance (Figure 3). Each corresponding PC was highly similar between the two sexes. We interpreted the first PC as reflecting an index of absolute diffusivity (Acosta-Cabronero et al., 2010), the second as an index of axonal density, and the third as a measure of the number of fiber orientations in a voxel. We then projected data from all other participants onto these three PCs (Figure 3).

Further details for the MRI acquisition, processing, and post-processing are presented in Supplementary material.

### 2.5 Statistical analyses

We performed all analyses separately by sex. We compared four groups (TD/TBI+, TD/TBI-, DBD/TBI+, DBD/TBI-) as to individual and family characteristics using two-sample *t-*tests for continuous variables and chi-squared tests for categorical variables with 2000 Monte Carlo simulations to calculate *p*-values. We conducted three sets of comparisons of WMM: (1) among children without a prior TBI, comparing DBD and TD groups; (2) among children with a prior TBI, comparing DBD and TD groups; (3) within DBD and TD groups, comparing children with and without a prior TBI. After having regressed out scanner, we used multivariate regression analyses to identify WMM differences, separately by bundle, non-adjusted and then adjusted separately for each covariate (CU MAP, ADHD, IQ, age, pubertal stage, ethnicity (0 = non-Hispanic White, 1 = other), and family income (0 = above $50,000, 1 = below $50,000). For bundles displaying significant group differences, we ran *post hoc* analyses comparing each WMM component.

## 3 Results

### 3.1 Sample characteristics

Sample characteristics of boys and girls are summarized in Tables 1, 2. Among the 749 boys, TBIs had been sustained by 7.75% of the DBD group and 2.59% of the TD group (χ ^2^= 9.859, *p* = 0.005). Among the 751 girls, TBIs had been sustained by 4.83% of DBD group and 2.07% of the TD group (χ^2^= 4.424, *p* = 0.048). Given that the TD group was defined not to include psychopathology, we reasoned that the excluded children who presented some psychopathology might have a higher risk of TBIs. To determine whether the elevations in risk of TBIs was specific to children presenting DBDs we compared them to all the other children in the ABCD sample. Among the boys who did not present DBDs (*n* = 5,631) 4.14% had sustained a TBI as compared to 7.36% among the full (i.e., before quality control, pre- and post-processing) boys DBD group (*X*^2^ = 12.442, *p* = 0.002). Among the girls who did not present DBDs (5,329), 3.0% sustained a TBI as compared to 4.26% among the full girls DBD group (*X*^2^ = 1.357, *p* = 0.206).

**Table 1:** Comparisons of boys presenting disruptive behavior disorders and their typically developing peers with and without traumatic brain injuries.

	**DBD (*****n*** **=** **400)**		**TD (*****n*** **=** **349)**		**Statistics**			
	**TBI (*n =* 31)**	**No TBI (*n =* 369)**	**TBI (*n =* 9)**	**No TBI (*n =* 340)**	**DBD/TBI- vs. DBD/TBI+**	**TD/TBI- vs. TD/TBI+**	**DBD/TBI- vs. TD/TBI-**	**DBD/TBI+ vs. TD/TBI+**
					***χ***^**2**^, ***p***			
**Pubertal stage** ^a^								
1	20 (66.7%)	241 (66.6%)	6 (66.7%)	224 (68.9%)	0.003, 1.000	0.262, 1.000	0.473, 0.778	0.231, 1.000
2	8 (26.7%)	96 (26.5%)	2 (22.2%)	79 (24.3%)				
3+	2 (6.6%)	25 (6.9%)	1 (11.1%)	22 (6.8%)				
**Race/ethnicity**								
Hispanic	5 (16.1%)	43 (11.7%)	3 (33.3%)	71 (20.9%)	1.293, 0.755	3.520, 0.311	11.893, **0.007**	3.097, 0.375
Non-Hispanic black	3 (9.7%)	60 (16.3%)	0 (0%)	52 (15.3%)				
Non-Hispanic white	19 (61.3%)	222 (60.2%)	6 (66.7%)	175 (51.5%)				
Other/Multi-racial	4 (12.9%)	44 (11.9%)	0 (0%)	42 (12.3%)				
**Combined household income** ^b^								
< $50,000	10 (36%)	102 (30.4%)	0 (0%)	75 (24.2%)	0.866, 0.684	7.420, **0.026**	6.894, **0.036**	11.496, **0.003**
$50,000-$100,000	9 (32%)	96 (28.6%)	0 (0%)	76 (24.5%)				
≥$100,000	9 (32%)	138 (41.0%)	8 (100%)	159 (51.3%)				
**Highest parent education**								
Some college or less	4 (12.9%)	41 (11.1%)	2 (22.2%)	46 (13.5%)	0.778, 0.755	1.819, 0.349	1.017, 0.592	1.275, 0.549
Associate degree	3 (9.7%)	57 (15.4%)	0 (0%)	49 (14.4%)				
Bachelor's and above	24 (77.4%)	271 (73.5%)	7 (77.8%)	245 (72.1%)				
**Parent marital status** ^c^								
Married/Living With Partner n (%)	19 (61%)	248 (68.5%)	8 (88.9%)	260 (77.8%)	0.683, 0.423	0.626, 0.679	7.680, **0.009**	2.422, 0.237
**Parent substance use** ^d^								
≥1 parent with substance use n (%)	12 (40%)	104 (29.1%)	1 (11.1%)	28 (8.4%)	1.557, 0.223	0.086, 1	48.314, ** < 0.001**	2.600, 0.214
Conduct problems	62.7 (8.58)	60.0 (8.87)	50 (0)	50 (0)	−1.705, 35.607, 0.097	-	21.657, 368, ** < 0.001**	8.270, 30, ** < 0.001**
Oppositional defiant problems	64.0 (9.39)	61.1 (8.16)	50 (0)	50 (0)	−1.701, 33.911, 0.098	-	26.076, 368, ** < 0.001**	8.318, 30, ** < 0.001**
NIH total cognition fully-corrected T-score	44.6 (10.1)	47.2 (11.3)	55.9 (13.0)	50.2 (11.9)	1.354, 35.673, 0.184	−1.307, 8.407, 0.226	−3.232, 623.55, **0.001**	−2.409, 11.086, **0.035**
CU MAP	1.02 (1.17)	0.887 (1.15)	−0.207 (0.07)	−0.214 (0.07)	−0.630, 35.054, 0.533	−0.283, 8.377, 0.784	18.303, 370.74, ** < 0.001**	5.811, 30.765, ** < 0.001**
ADHD score	65.1 (9.23)	58.7 (8.22)	50 (0)	50 (0)	−3.734, 34.116, ** < 0.001**	-	20.352, 368, ** < 0.001**	9.109, 30, ** < 0.001**
Mean age (sd)	9.95 (0.64)	9.94 (0.61)	10.35 (0.63)	9.95 (0.63)	−0.102, 34.691, 0.919	−1.899, 8.425, 0.092	−0.253, 698.34, 0.800	−1.685, 13.228, 0.115

^a^1 boy in the disruptive behavior disorder (DBD) group with traumatic brain injury (TBI), 7 boys in the DBD group without a TBI, and 15 boys in TD group without TBI had missing pubertal stage data. ^b^3 boys presenting DBD with a TBI, 33 boys presenting DBD without a TBI, 1 TD boy with a TBI, and 30 TD boys without a TBI had missing combined household income data. ^c^Parents of 7 DBD boys without a TBI and 6 TD boys without a TBI had missing marital status data. ^d^Parents from 1 DBD boy with TBI, 12 DBD boys without TBI, and 5 TD boys without a TBI had missing data on substance use problems. DBD, disruptive behavior disorders; TBI, traumatic brain injury; CU MAP, callous-unemotional maximum a posteriori scores; ADHD, attention deficit disorder; NIH, National Institutes of Health. Statistically significant *p*-values have been highlighted in bold.

**Table 2:** Comparisons of girls presenting disruptive behavior disorders and their typically developing peers with and without traumatic brain injuries.

	**DBD (*****n*** **=** **269)**		**TD (*****n*** **=** **482)**		**Statistics**			
	**TBI (*****n** =* **13)**	**No TBI (*****n** =* **256)**	**TBI (*****n** =* **10)**	**No TBI (*****n** =* **472)**	**DBD/TBI- vs. DBD/TBI**+	**TD/TBI-vs. TD/TBI**+	**DBD/TBI-vs. TD/TBI-**	**DBD/TBI**+ **vs. TD/TBI**+
					*χ^2^**, p***			
**Pubertal stage** ^a^								
1	3 (25%)	72 (29.3%)	3 (30%)	154 (33.6%)	1.500, 2, 0.513	1.892, 0.401	6.417, **0.043**	0.105, 1.000
2	1 (8.3%)	50 (20.3%)	1 (10%)	118 (25.8%)				
3+	8 (66.7%)	124 (50.4%)	6 (60%)	186 (40.6%)				
**Race/ethnicity** ^b^								
Hispanic	3 (23.1%)	36 (14.1%)	0 (0%)	95 (20.2%)	1.123, 0.788	3.386, 0.374	6.022, 0.104	2.654, 0.555
Non-Hispanic black	1 (7.7%)	30 (11.7%)	1 (10%)	67 (14.2%)				
Non-Hispanic white	8 (61.5%)	157 (61.3%)	8 (80%)	254 (53.9%)				
Other/Multi-racial	1 (7.7%)	33 (12.9%)	1 (10%)	55 (11.7%)				
**Combined household income** ^c^								
< $50,000	5 (45.4%)	73 (31.1%)	1 (10%)	93 (21.5%)	1.374, 0.508	6.458, **0.026**	7.413, **0.029**	8.639, 0.010
$50,000-$100,000	3 (27.3%)	59 (25.1%)	0 (0%)	122 (28.2%)				
≥$100,000	3 (27.3%)	103 (43.8%)	9 (90%)	217 (50.3%)				
**Highest parent education**								
Some college or less	0 (0%)	29 (11.3%)	0 (0%)	52 (11%)	6.887, **0.036**	3.091, 0.227	0.167, 0.921	4.915, 0.053
Associate degree	5 (38.5%)	35 (13.7%)	0 (0%)	60 (12.7%)				
Bachelor's and above	8 (61.5%)	192 (75%)	10 (100%)	360 (76.3%)				
**Parent marital status** ^d^								
Married/living with partner^e^ n (%)	8 (61.5%)	173 (67.6%)	9 (90%)	369 (78.8%)	0.205, 0.775	0.736, 0.496	11.165, **0.002**	2.375, 0.164
**Parent substance use** ^e^								
≥1 parent with substance use n (%)	6 (46.2%)	80 (32.1%)	1 (10%)	54 (11.8%)	1.102, 0.374	0.031, 1	43.245, ** < 0.001**	3.490, 0.088
Conduct problems	64.1 (8.89)	58.2 (8.44)	50 (0)	50 (0)	−2.338, 13.121, **0.036**	-	15.509, 255, ** < 0.001**	5.708, 12, ** < 0.001**
Oppositional defiant problems	64.8 (8.58)	59.8 (7.52)	50 (0)	50 (0)	−2.096, 12.952, 0.056	-	20.780, 255, ** < 0.001**	6.238, 12, ** < 0.001**
NIH total cognition fully-corrected T-score	46.8 (12.2)	47.7 (11.4)	49.6 (8.43)	50.4 (10.9)	0.240, 13.212, 0.814	−0.293, 8.580, 0.776	−2.940, 449.34, **0.003**	−0.615, 19.997, 0.546
CU MAP	0.174 (1.04)	0.360 (1.19)	−0.485 (0.04)	−0.515 (0.07)	0.627, 13.648, 0.541	−2.317, 10.021, **0.043**	11.759, 255.85, ** < 0.001**	2.280, 12.048, **0.042**
ADHD score	62.8 (8.21)	56.9 (7.46)	50 (0)	50 (0)	−2.549, 13.025, **0.024**	-	14.835, 255, ** < 0.001**	5.639, 12, ** < 0.001**
Mean age (SD)	10.11 (0.64)	9.89 (0.63)	10.40 (0.62)	10.00 (0.60)	−1.206, 13.233, 0.249	−2.053, 9.359, 0.070	−2.177, 496.57, **0.030**	−1.106, 19.812, 0.282

^a^1 girl with disruptive behavior disorder (DBD) with a traumatic brain injury (TBI), 10 DBD girls without TBIs, and 14 TD without TBIs had missing pubertal stage data. ^b^1 TD girl without a TBI had missing race/ethnicity data. ^c^Parents of 2 DBD girls with a TBI, 21 DBD girls without a TBI, and 40 TD girls without a TBI had missing combined household income data. ^d^Parents of 4 TD girls without TBIs had missing marital status data. ^e^Parents from 7 DBD girls without a TBI and 15 TD girls without a TBI had missing data on substance use problems. DBD, disruptive behavior disorders; TBI, traumatic brain injury; CU MAP, callous-unemotional maximum a posteriori scores; ADHD, attention deficit disorder. Statistically significant *p*-values have been highlighted in bold.

Children presenting DBDs, their parents and families, differed from TD children, parents and families, obtaining higher scores for conduct problems, oppositional defiant problems, CU, ADHD, lower IQ, lower family income, fewer living with two parents, and more had a family member presenting substance misuse. Among the DBD boys, there was only one difference between those with and without TBIs; previously injured boys obtained higher ADHD scores. Among the TD boys, only family income distinguished those with and without TBIs. Among the DBD girls, those who had sustained a TBI differed from those who had not by having parents with a lower level of education, higher scores for conduct problems and for ADHD. Among TD girls, those with TBIs differed from those without by coming from families with higher income and by obtaining higher CU scores.

### 3.2 Among children without TBIs, are there WMM differences between those presenting DBDs and TD children?

As presented in Table 3, among boys who had not experienced TBIs, those with DBDs as compared to TD boys displayed a multivariate difference of WMM in the left CST, robust to adjustment for age, ethnicity, and family income, but not for CU, ADHD, IQ, or pubertal stage, and in the CC genu, robust to adjustment for IQ, age, pubertal stage, ethnicity, and family income, but not CU and ADHD. In *post hoc* analyses, no differences were detected in the three components of WMM of the CST. In the CC genu, absolute diffusivity was lower and axonal density was higher in DBD boys compared to TD boys.

**Table 3:** Among children who have not experienced a traumatic brain injury, comparisons of white matter microstructure between boys and girls with disruptive behavior disorders and typically developing boys and girls, with adjustment for callous-unemotional traits, attention deficit hyperactivity disorder, IQ, age, pubertal stage, ethnicity, and family income.

	**Multivariate model** ***p*****-values**								
**Bundle**	**Non-adjusted**	**Adjusted for**							**Differences in components**
	***p*****-values (**η^2^**)**	**CU MAP**	**ADHD**	**IQ**	**Age**	**Pubertal stage**	**Ethnicity**	**Family income**	
**Boys (*****n** =* **709)**									
**Left**									
UF	0.3346								
IFOF	0.9611								
CG	0.9893								
ILF	0.8612								
CST	**0.0366** (0.01)	0.2511	0.1532	0.0813	**0.0354**	0.0764	**0.0211**	**0.0373**	None
**Right**									
UF	0.5173								
IFOF	0.9762								
CG	0.9219								
ILF	0.6080								
CST	0.2058								
**CC**									
Genu	**0.0077** (0.02)	0.3117	0.0880	**0.0293**	**0.0080**	**0.0097**	**0.0051**	**0.0074**	Absolute diffusivity: TD>DBD Axonal density: TD < DBD
Body	0.5306								
Splenium	0.9581								
**Girls (*****n** =* **728)**									
**Left**									
UF	0.0585								
IFOF	**0.0257** (0.01)	0.1329	**0.0277**	**0.0388**	**0.0271**	**0.0275**	**0.0284**	**0.0480**	Axonal density: TD < DBD
CG	0.6011								
ILF	0.7111								
CST	0.7171								
**Right**									
UF	0.1683								
IFOF	**0.0118** (0.02)	0.2511	0.2249	**0.0173**	**0.0115**	**0.0084**	**0.0160**	**0.0133**	Axonal density: TD < DBD
CG	0.3848								
ILF	**0.0428** (0.01)	0.1644	0.2937	0.1566	**0.0306**	0.0882	0.0572	**0.0101**	Axonal density: TD>DBD
CST	0.6183								
**CC**									
Genu	0.0560								
Body	**0.0214** (0.01)	**0.0156**	0.1504	0.0651	**0.0249**	**0.0304**	**0.0120**	0.0535	Absolute diffusivity: TD>DBD
Splenium	0.2957								

TD, typically developing; DBD, Disruptive Behavior Disorders; UF, Uncinate Fasciculus; IFOF, Inferior Fronto-Occipital Fasciculus; CG, Cingulum; ILF, Inferior Longitudinal Fasciculus; CST, Corticospinal Tract; CC, Corpus Callosum; CU MAP, Callous Unemotional maximum a posteriori scores; ADHD, Attention Deficit/Hyperactivity Disorder. η^2^, Generalized eta squared. Statistically significant *p*-values have been highlighted in bold.

Among girls without TBIs, those presenting DBDs, as compared to TD girls, displayed a multivariate difference of WMM in the left IFOF, robust to adjustment for all covariates except CU, in the right IFOF, robust to adjustment for all covariates except CU and ADHD, in the right ILF, robust to adjustment only for age and family income, and in the body of the CC, robust to adjustment for CU, age, pubertal stage, and ethnicity. *Post hoc* analyses revealed that DBD girls showed higher axonal density in the left and right IFOF, lower axonal density in the right ILF, and lower absolute diffusivity in the CC body.

### 3.3 Among children with TBIs, are there WMM differences between those presenting DBDs and TD children?

As presented in Table 4, among boys with TBIs, those with DBDs as compared to TD boys displayed a multivariate difference of WMM in the left CST, robust to adjustment for CU, IQ, pubertal stage, and ethnicity. In *post hoc* analyses, no differences in the three components of WMM were detected.

**Table 4:** Among children who have experienced a traumatic brain injury, comparisons of white matter microstructure between boys and girls with disruptive behavior disorders and typically developing boys and girls, with adjustment for callous-unemotional traits, attention deficit hyperactivity disorder, IQ, age, pubertal stage, ethnicity, and family income.

		**Multivariate model** ***p*** **values**							
**Bundle**	**Non-adjusted**	**Adjusted for**							**Differences in components**
	***p*****-values (**η^2^**)**	**CU MAP**	**ADHD**	**IQ**	**Age**	**Pubertal stage**	**Ethnicity**	**Family income**	
**Boys (*****n** =* **40)**									
**Left**									
UF	0.3374								
IFOF	0.1219								
CG	0.2818								
ILF	0.0910								
CST	**0.0263** (0.22)	**0.0185**	0.1104	**0.0313**	0.0568	**0.0397**	**0.0275**	0.0863	None
**Right**									
UF	0.7966								
IFOF	0.6124								
CG	0.5597								
ILF	0.7011								
CST	0.2385								
**CC**									
Genu	0.6557								
Body	0.3704								
Splenium	0.1050								
**Girls (*****n** =* **23)**									
**Left**									
UF	0.5464								
IFOF	0.4380								
CG	0.9113								
ILF	0.8297								
CST	0.4828								
**Right**									
UF	**0.0251** (0.38)	**0.0058**	**0.0111**	**0.0375**	**0.0375**	**0.0048**	**0.0352**	0.1657	Axonal density: TD < DBD
IFOF	0.0963								
CG	0.3728								
ILF	0.3205								
CST	0.5709								
**CC**									
Genu	0.3739								
Body	0.4913								
Splenium	0.6331								

TD, typically developing; DBD, Disruptive Behavior Disorders; UF, Uncinate Fasciculus; IFOF, Inferior Fronto-Occipital Fasciculus; CG, Cingulum; ILF, Inferior Longitudinal Fasciculus; CST, Corticospinal Tract; CC, Corpus Callosum; CU MAP, Callous Unemotional maximum a posteriori scores; ADHD, Attention Deficit/Hyperactivity Disorder. η^2^, Generalized eta squared. Statistically significant *p*-values have been highlighted in bold.

Among girls with TBIs, those presenting DBDs, as compared to the TD, showed a multivariate difference in WMM in the right UF, robust to adjustment for all covariates except family income. In *post hoc* analyses, the UF of DBD girls showed higher axonal density.

### 3.4 Among TD boys and girls, do those who have sustained TBIs show WMM differences from those with no TBIs?

Among both TD boys and girls, there were no significant differences in WMM between those with and without TBIs in any of the 13 bundles studied.

### 3.5 Among boys and girls presenting DBDs, do those who have sustained TBIs differ from those with no TBIs?

As presented in Table 5, among boys presenting DBDs, those with prior TBIs compared to those without displayed a multivariate difference of WMM in the left CST, robust to adjustment for CU, ADHD, age, ethnicity, and family income, and in the right ILF, robust to adjustment for all covariates except ADHD and family income. *Post hoc* analyses revealed that DBD boys who had sustained TBIs showed higher axonal density in the left CST and higher absolute diffusivity in the right ILF, compared to DBD boys without TBIs.

**Table 5:** Among boys and girls presenting disruptive behavior disorders, comparisons of white matter microstructure between those who had and had not sustained a traumatic brain injury, with adjustment for callous-unemotional traits, attention deficit hyperactivity disorder, IQ, age, and pubertal stage, ethnicity, and family income.

		**Multivariate model** ***p*****-values**							
**Bundle**	**Non-adjusted**	**Adjusted for**							**Differences in components**
	***p*****-values (**η^2^**)**	**CU MAP**	**ADHD**	**IQ**	**Age**	**Pubertal stage**	**Ethnicity**	**Family income**	
**Boys (*****n** =* **400)**									
**Left**									
UF	0.4662								
IFOF	0.8035								
CG	0.7851								
ILF	0.2472								
CST	**0.0392** (0.02)	**0.0349**	**0.0236**	0.1085	**0.0390**	0.0813	**0.0394**	**0.0231**	Axonal density: TBI+>TBI-
**Right**									
UF	0.6261								
IFOF	0.5252								
CG	0.5661								
ILF	**0.0241** (0.02)	**0.0262**	0.0656	**0.0240**	**0.0230**	**0.0174**	**0.0244**	0.2794	Absolute diffusivity: TBI+>TBI-
CST	0.9050								
**CC**									
Genu	0.9735								
Body	0.3543								
Splenium	0.6314								
**Girls (*****n** =* **269)**									
**Left**									
UF	0.9623								
IFOF	0.6460								
CG	0.9446								
ILF	0.8010								
CST	0.5054								
**Right**									
UF	0.3650								
IFOF	0.4694								
CG	0.8951								
ILF	0.4452								
CST	0.6829								
**CC**									
Genu	**0.0369** (0.03)	**0.0358**	**0.0249**	**0.0483**	**0.0436**	**0.0052**	**0.0363**	0.2069	Absolute diffusivity: TBI+>TBI-
Body	0.3849								
Splenium	0.7792								

TD, typically developing; DBD, Disruptive Behavior Disorders; UF, Uncinate Fasciculus; IFOF, Inferior Fronto-Occipital Fasciculus; CG, Cingulum; ILF, Inferior Longitudinal Fasciculus; CST, Corticospinal Tract; CC, Corpus Callosum; CU MAP, Callous Unemotional maximum a posteriori scores; ADHD, Attention Deficit/Hyperactivity Disorder. η^2^, Generalized eta squared. Statistically significant *p*-values have been highlighted in bold.

Among girls presenting DBDs, there was only one significant difference between those who had and who had not sustained TBIs that was observed in the genu of the CC, robust to adjustment for all covariates except family income. *Post hoc* analyses revealed that DBD girls who had sustained TBIs showed higher absolute diffusivity in the CC genu compared to DBD girls without TBIs.

### 3.6 Adjusting for multiple comparisons

Given the large number of statistical comparisons, we performed a Benjamini–Hochberg correction for each set of analyses (i.e., a separate one for the analyses presented in Tables 3–5). No comparisons survived corrections for statistical comparisons.

## 4 Discussion

In the present study, we found that greater proportions of boys and girls with DBDs than TD boys and girls had sustained at least one TBI by age 10, consistent with previous findings (Guberman et al., 2020b). Factors associated with increased risk of TBIs may differ in the two groups. Children with DBDs are more likely than TD children to have experienced harsh parenting and/or maltreatment (Jaffee et al., 2005) and to engage in risky behaviors, such as physical fighting. By contrast, TD children may be more likely to participate in organized sports involving heightened risk of TBIs. The prevalence of TBIs was higher among boys than girls with DBDs, and similar in TD boys and girls. There is a paucity of research on girls with DBDs, although existing studies identify few sex differences in developmental trajectories (Freitag et al., 2018). While CU traits are lower in girls with DBDs they have similar brain correlates (Pardini et al., 2012). However, some characteristics that could be associated with TBIs have been reported to be elevated in boys such as risky decision making (Sidlauskaite et al., 2018), impulsivity (Hartung et al., 2002), and risky behaviors leading to premature death (Kratzer and Hodgins, 1997).

Among children with DBDs, those without TBIs showed alterations in WMM relative to sex-matched TD children. Within the DBD group, those who had sustained TBIs also showed differences in WMM relative to the non-injured. Disentangling alterations of WMM associated with TBIs from those associated with DBDs, CU, and ADHD provides information useful to the treatment of each comorbid condition and will further understanding of the etiology of these common childhood disorders that often have negative life-long consequences.

### 4.1 Children who had not sustained a TBI

In the present study, among children who had not sustained TBIs, alterations of WMM were observed among those with DBDs relative to those who were TD. Analyses performed within sex showed different alterations, all of which were related to ADHD, CU or both. Among boys without TBIs, those with DBDs relative to the TD group showed differences in the left CST and the genu of the CC. Previous studies that did not exclude participants who had sustained TBIs identified differences in the CST and the CC genu among those with DBDs comorbid with ADHD relative to those with DBDs alone and higher fractional anisotropy and lower mean/radial diffusivity among boys with conduct disorder and CU, but not among those with conduct disorder alone (Puzzo et al., 2018). Further, a systematic review and meta-analysis reported that children/adolescents with ADHD showed alterations in the CST believed to be related to motor disinhibition or dysregulation of dopamine in downstream pathways (Hamilton et al., 2008).

Among girls who had not experienced TBIs, those presenting DBDs displayed differences in the left and right IFOF, the right ILF, and the body of the CC. Only the difference in the CC was robust to adjustment for CU. These results are consistent with previous studies of females with current or past conduct disorder, that reported alterations in the CC and lower fractional anisotropy and axial diffusivity in the anterior/superior corona radiata, ILF and IFOF (Budhiraja et al., 2017).

Both girls and boys presenting with DBDs without TBIs showed differences relative to TD children in the CC, which has long been associated with antisocial behavior among adult males (Raine et al., 2003). Alterations of the CC are believed to underlie problems in emotional regulation, motor coordination, motor planning, executive functions, and impulsivity (Schutter and Harmon-Jones, 2013). Corpus callosum axial diffusivity has been shown to mediate the association between CU and impulsive responses to emotional faces (Rogers et al., 2019). In the present study, among girls who had not sustained TBIs, those with DBDs displayed differences in the right ILF and bilateral IFOF that connect the posterior temporal and occipital lobes, including visual and auditory association areas, to the prefrontal cortex (Catani et al., 2013), and the ILF to the amygdala (Fox et al., 2008). Alterations of these tracts are believed to be related to impairments in emotion processing (Herbet et al., 2018) and goal-oriented behavior (Conner et al., 2018). Among children/adolescents with DBDs, these regions show alterations of gray matter and functioning. A recent study of the ABCD sample reported that children with DBDs with and without CU, compared to TD children, displayed smaller amygdala volumes bilaterally (Waller et al., 2020). A European multi-center study of 118 children presenting DBDs and 89 healthy children found proactive aggression was related to increased functional coupling between the amygdala and precuneus, reactive aggression to amygdala-left lateral cortex hyperconnectivity, and callousness to right prefrontal cortex-right precentral gyrus hyperconnectivity (Werhahn et al., 2021).

### 4.2 Children who had sustained a prior TBI

Among boys with prior TBIs, those with DBDs showed only one difference relative to TD boys that was in the left CST, as was the case for boys without prior TBIs. This difference did not survive adjustment for ADHD. This finding is consistent with a prior report of differences in WMM in the CST among male TBI patients with ADHD compared to healthy children (Hamilton et al., 2008). Among girls with prior TBIs, those presenting DBDs displayed only one difference relative to TD girls that was in the right UF, robust to adjustment for all covariates except family income. The UF has been previously associated with DBDs (Waller et al., 2017; Bolhuis et al., 2019; Rogers et al., 2019), as it connects neural regions involved in behavioral control, such as the orbitofrontal cortex, with areas involved in threat perception, such as the amygdala (Fox et al., 2015).

### 4.3 Children presenting DBDs or TD with and without a history of TBIs

We observed striking differences when examining WMM of children with and without TBIs within the DBD and TD groups. Among TD children, no significant differences were detected in any WMM bundles between those with and without prior TBIs. Given the variability in pre-injury factors and post-TBI consequences, group comparisons may only detect shared abnormalities (Guberman et al., 2022), such that alterations in WMM may only be detected within a group displaying a common underlying structural abnormality, such as children with DBDs. Consistent with this idea, a prior study found that in the Philadelphia Neurodevelopmental Cohort, children who had sustained a TBI, relative to healthy children, displayed differences in deep white matter, but when compared to children who were matched for levels of psychopathology, no differences were detected (Stojanovski et al., 2019).

### 4.4 Development of WMM

The use of our multidimensional approach to measure WMM provided information regarding the nature of the abnormalities not available from previous studies. Across most DBD/TD comparisons (except right ILF in DBD girls without TBIs), axonal density scores were higher among children presenting DBDs. These results could be interpreted as suggesting accelerated development of WMM. This hypothesis is consistent with a recent study reporting increases in apparent fiber density across development among healthy children (Genc et al., 2020). In these same comparisons, other bundles consistently displayed lower absolute diffusivity in the DBD group. If WMM development is accelerated among children with DBDs, lower absolute diffusivity may reflect other processes, such as the increased presence of neurofibrils, microglia, and myelin from oligodendrocytes (Acosta-Cabronero et al., 2010), perhaps to support the higher number of axons. Longitudinal studies of healthy children have shown concurrent decreases in axial and radial diffusivity across development (Lebel and Beaulieu, 2011). The observation of these concurrent microstructural processes, occasionally present simultaneously in the same bundle (for example boys' CC genu, Table 3), reveals a particular strength of the measures of WMM used in the present study. The principal components analyses yielded three orthogonal (non-correlated) components of WMM, a distinct advantage over the highly-correlated tensor-based measures.

Unlike the findings from comparisons of DBD and TD groups, within the DBD group, absolute diffusivity scores were higher among children who had sustained TBIs relative to the non-injured. This finding may reflect injury-related loss of myelin and other supporting structures, although this conclusion requires histological validation. Among boys with DBDs, those with TBIs displayed higher axonal density scores than those without. This result is surprising, and runs counter to prior research (Guberman et al., 2020a). However, this effect was lost when adjusting for pubertal stage and IQ, suggesting possibly that differences in WMM maturation may be partly responsible, even if pubertal stage and IQ were similar in the two groups.

### 4.5 Ethnicity and family income

Ethnicity differed little in DBD and TD boys and girls, with and without TBIs. In comparisons of WMM, ethnicity modified the significance of only one result. Among girls who had not experienced TBIs, the difference between the DBD and TD groups in the right ILF did not survive adjustment for ethnicity. The TD boys and girls came from families reporting slightly higher income than the DBD children. Family income played no role in comparisons of DBD and TD children who did not sustain TBIs. By contrast, in comparisons of DBD children who had sustained TBIs to both TD children with TBIs and to DBD children without TBIs, several differences lost significance when models were adjusted for family income. Low family income may index a number of factors that directly or indirectly impact the child's neural development and their risk of sustaining TBIs, such as harsh parenting, neglect, and monitoring of child behavior.

### 4.6 Clinical implications

Children at risk for TBIs include those presenting conduct problems and/or inattention-hyperactivity, some of whom engage in aggressive behavior, those who have experienced a prior TBI, and those experiencing maltreatment and/or neglect and/or age-inappropriate parental monitoring. Nurse visitation programs in the years following birth could be modified to include assessments of toddlers' impulsivity, risk taking, obedience, and aggressive behavior and parents' harsh and inappropriate punishment, neglect, and age-appropriate monitoring of the child's behavior. These same child and parent characteristics could be assessed by pre-school staff and elementary school teachers. Ideally, interventions could be provided to children and/or parents presenting characteristics that elevate the risk of TBIs. Adding components to treatment programs for conduct problems and ADHD that focus on impulsivity and risk taking has the potential to prevent TBIs. Effectively eliminating maltreatment and neglect could also prevent TBIs. The effectiveness of treatments for childhood TBIs would be improved by taking account of the child's and the family's pre-injury characteristics and by implementing strategies to prevent further TBIs. Consistent with the current findings, previous research has found that after taking account of either inattention-hyperactivity or conduct problems, children who sustained a TBI by age 10, were three times more likely than children who had not sustained a TBI to experience at least one more TBI before age 18 years (Guberman et al., 2020b).

### 4.7 Limitations and strengths

The present conclusions must be considered in light of some methodological limitations. By design, the ABCD Study excluded severe TBIs. Although information about the number of TBIs sustained by participants and ages when the injuries occurred was not available, the commitment of parents and children required by the ABCD Study is considerable, possibly discouraging families whose children had sustained more severe and symptomatic head injuries from participating. This likely underrepresentation of more severe and/or symptomatic TBIs is a limitation of our study. Even among those with DBDs, the prevalence of TBIs was lower than that of 12% in the general population (Frost et al., 2013). The number of TD participants who had sustained a TBI was particularly low. In the within sex comparisons of DBD and TD groups with a history of TBIs, this low number of TBI cases could have led to issues with homogeneity of variance due to unequal sample sizes. Further, in these comparisons, effect sizes, as measured by the Generalized η^2^ statistic were found to be larger than in other analyses. This statistic is believed to overestimate the true effect size, and this bias decreases with increasing sample size (Mordkoff, 2019). Hence, it is possible that the differences in effect size observed in this set of analyses appeared larger as a consequence of their smaller sample size.

The decision to adjust for several covariates while taking into account the relatively low number of participants in some of the subgroups that were compared led us to favor simpler models without interaction terms. Opting for simpler models also meant running more of them, a limitation that was compounded in part by our liberal bundle selection procedure. We focused on any bundles that were previously reported to be associated with DBDs, even if they had only been reported on only once. This decision was deliberate: given that our project is the first to study white matter structure in boys and girls with DBDs with prior TBIs, our approach to bundle selection, although partly hypothesis driven, was mostly exploratory. As a result of these methodological choices, a large number of statistical comparisons were performed. Several efforts were taken to reduce the number of potential comparisons, such as our PCA and our multivariate regression approaches which reduced the number of comparisons by a factor of 2 and 3 respectively, and the fact that we only adjusted for covariates in models with significant main effects of group. Nonetheless, to remedy the expected increase in Type 1 error, we decided to be conservative in our adjustment for multiple comparisons. Future work with larger numbers of DBD and TD children with TBIs will need to consider running fewer, more complex models of WMM in a targeted set of tracts, adjusting for all relevant covariates together and incorporating interaction terms.

Another potential limitation is the possibility that controlling for conditions that are likely to be highly comorbid with DBDs and/or TBIs may have introduced multicollinearity into our models, and could have led to adjusted effects that are difficult to interpret and unnaturalistic (Miller and Chapman, 2001). Finally, information on maltreatment – which is more common among children with than without DBDs (Jaffee et al., 2005) and a cause of TBIs especially in young children (Duhaime and Christian, 2019) – was not available.

Strengths of the study include the relatively large sample, especially of females, who presented DBDs. Another strength was the age of participants that likely precluded substance use. The study employed novel modeling, tractography, tractometry, and statistical approaches to measure WMM that are robust to the limitations of more conventional analyses and that extract more exhaustive information. Utilizing tractography robust to partial volume effects and a highly reproducible automatic bundle clustering algorithm increases the accuracy of bundle reconstructions and hence the localization of the reported effects. The use of a modeling technique robust to crossing fibers and a data recombination approach to create more biologically-interpretable measures of WMM allowed us to make more fine-grained interpretations of the obtained effects.

### 4.8 Conclusion

Children with DBDs are at increased risk relative to healthy children to sustain TBIs. Those who have not sustained TBIs show alterations of WMM relative to TD children, while those who have sustained such injuries show additional alterations. Furthering understanding of the etiology and improving treatment of DBDs will require disentangling alterations of WMM that are specific to girls and boys, with and without CU, ADHD, and TBIs. Additionally, it is critical to determine the temporal associations of DBD onset and persistence with TBIs. Assessing pre-injury characteristics of children who have sustained TBIs could contribute to personalizing treatment.

## Data availability statement

Publicly available datasets were analyzed in this study. This data can be found at: https://abcdstudy.org/consortium_members/. The ABCD data used in this report came from https://dx.doi.org/10.15154/1503209.

## Ethics statement

Ethical approval was not required for the studies involving humans because this study used the open-access ABCD Study dataset. Ethics approval was obtained by the dataset's administrators previously, and was not required for the present manuscript. The studies were conducted in accordance with the local legislation and institutional requirements. Written informed consent for participation in the present study was not required from the participants or the participants' legal guardians/next of kin in accordance with the national legislation and institutional requirements because consent for study participation was obtained by the dataset's administrators previously, and was not required for the present manuscript.

## Author contributions

GG: Conceptualization, Data curation, Formal analysis, Investigation, Methodology, Software, Validation, Visualization, Writing – original draft, Writing – review & editing. GT: Formal analysis, Resources, Software, Writing – review & editing. SH: Data curation, Funding acquisition, Project administration, Resources, Software, Writing – review & editing. AP: Conceptualization, Funding acquisition, Resources, Supervision, Writing – review & editing. MD: Investigation, Methodology, Resources, Software, Supervision, Writing – review & editing. SH: Conceptualization, Funding acquisition, Project administration, Supervision, Writing – original draft, Writing – review & editing.
