# Supplementary material for: White matter microstructure, traumatic brain injury, and disruptive behavior disorders in girls and boys
Source: Front Neurosci. 2024 Jul 19;18:1391407. doi: 10.3389/fnins.2024.1391407 (PMC11295658; doi:10.3389/fnins.2024.1391407)
Supplement: Supplementary file 1 [file Data_Sheet_1.docx]

**White matter microstructure, traumatic brain injury, and disruptive behavior disorders in girls and boys**

**Guido I. Guberman, Guillaume Theaud, Samuel W. Hawes, Alain Ptito, Maxime Descoteaux, Sheilagh Hodgins**

**Supplemental Information**

**METHODS AND MATERIALS**

**DWI scans**

MRI scans were acquired across 21 sites, using 28 different scanners. Details about the acquisition protocols are outlined in Casey et al. (2018). Multi-shell dMRI scans had 96 diffusion-weighted directions, with 6 directions of b=500 s/mm^2^, 15 of b=1000s/mm^2^, 15 of b=2000s/mm^2^, and 60 of b=3000s/mm^2^. The b=2000s/mm^2^ shell, which had directions that were collinear with the b=1000s/mm^2^ shell was excluded from processing. In addition, scans had 6 or 7 b=0s/mm^2^ images, depending on scanner type. A reverse b0 image was included for each participant to correct for EPI distortions.

**Processing.** During processing we selected white matter seeding given that this method yields fuller bundle reconstructions (Gauvin, 2016; Rheault, 2020). In comparison, white-gray matter interface seeding is known to yield thinner tracts, especially when reconstructing long bundles, given that the chances of a streamline encountering an obstacle causing premature termination increases as a function of the number of voxels it must traverse.

We selected 12 seeds-per-voxel, to obtain approximately 2 million streamlines across the brain. This target number was selected so as to achieve adequate “saturation” of bundles, that is, capture their full spatial extent (Gauvin, 2016; Rheault, 2020). One way of calculating bundle saturation is by comparing the volume occupied by a bundle against the total number of streamlines in the full tractogram. Although there is no agreed-upon consensus for the optimal total number of streamlines, and different bundles may have different saturation points, preliminary evidence suggests that several of the major white matter bundles appear to reach a stable volume around 2 million streamlines (Rheault, 2020).

We used the b=0, 500, and 1000 shells to perform tensor fitting, and the b=0 and 3000 shells to perform Constrained Spherical Deconvolution (CSD) (Descoteaux, Deriche, Knosche, & Anwander, 2009; Tournier, Calamante, & Connelly, 2007). We fixed the fiber-response functions for the entire sample by first calculating it on a subset of 71 randomly-selected typically developing children, using voxels with high (>0.70) fractional anisotropy (FA). The fiber response function was set to 17*10^-4^, 4*10^-4^, 4*10^-4^ (17, 4, 4 in Tractoflow using the *manual_frf* option). Lastly, we created tractograms using a probabilistic particle-filtering tractography algorithm (Girard, Whittingstall, Deriche, & Descoteaux, 2014). This tractography technique consists in informing stopping criteria using partial volume estimation maps computed from the high resolution T1-weighted images that were transformed to DWI space during processing with Tractoflow. PFT can achieve tractography results that are less biased by length, shape, size, and position (Girard et al. 2014). To increase the anatomical validity, this algorithm also imposes the anatomical constraint that streamlines obligatorily terminate in gray matter.

**Post-processing.** *RBX-flow* uses Nextflow (Di Tommaso et al., 2017; Kurtzer, Sochat, & Bauer, 2017), a parallelizable programming language, to implement RBX, a multi-atlas multi-parameter version of Recobundles (Garyfallidis et al., 2018) with label fusion. Technical details about this pipeline can be found in (Rheault, 2020). Briefly, this technique consists in first simplifying a whole-brain tractogram using a small number of clusters, grouping together streamlines by shape and similarity. Bundle “models”, manual segmentations performed by expert neuroanatomists, are then used to identify clusters that match the models with respect to a similarity measure based on shape and orientation. This approach is repeated several times, using multiple models, and multiple thresholds of similarity. Streamlines are given a score, determining, for every bundle, how often each streamline was assigned to each model. For instance, if we wish to extract 15 bundles and use 5 models for each, and test 10 different thresholds, every streamline will have a score on 50 (5 models x 10 thresholds) for each one of our 15 bundles. Streamlines can then be assigned to the bundle for which they obtained the highest score. Prior work has shown that manual segmentations have inherent variability (Rheault, De Benedictis, et al., 2020). By using a clustering algorithm this approach can provide more consistent bundle segmentations, and by using a multi-atlas approach, it can minimize the variability introduced by manual segmentations.

**Figure S1.** Illustration of principal components analysis procedure.


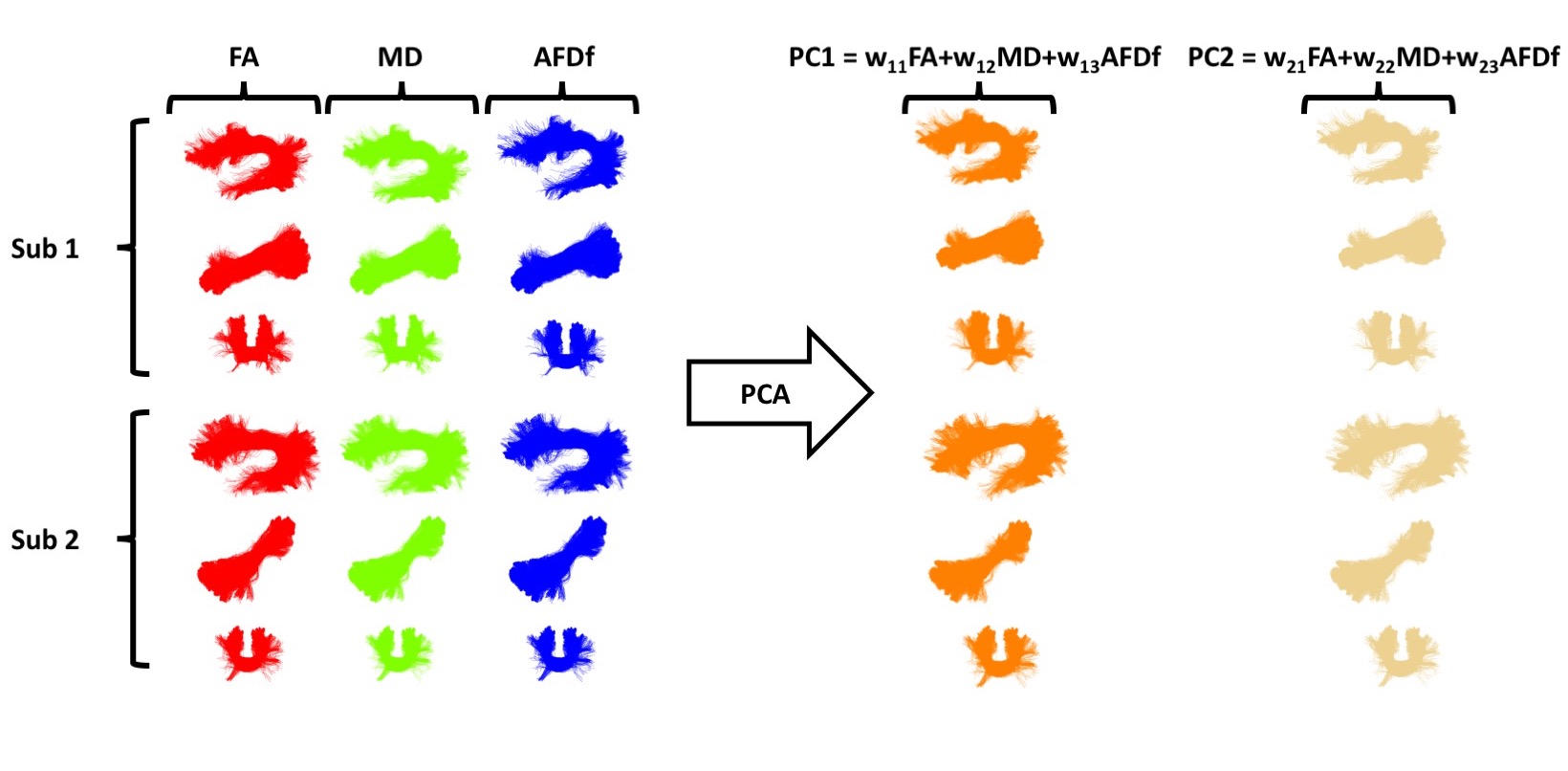


**Figure S1.** Schematic illustration of the PCA procedure. We performed PCA on the concatenated set of standardized diffusion measures across subjects (“sub”) and bundles. This approach yielded principal components (”PC”), linear combinations of every diffusion measure. These linear combinations are represented by the equations on the top right, where every measure is multiplied by a weight (“w”). These weights (also called loadings) are selected so that PCs account for the most variance across measures. Every subject’s bundle has a value (referred to as a “score”) for every PC. FA: Fractional Anisotropy; MD: Mean diffusivity; AFDf: Apparent Fiber Density along fixels.

**References**

Acosta-Cabronero, J., Williams, G. B., Pengas, G., & Nestor, P. J. (2010). Absolute diffusivities define the landscape of white matter degeneration in Alzheimer's disease. *Brain, 133*(Pt 2), 529-539. doi:10.1093/brain/awp257

Casey, B. J., Cannonier, T., Conley, M. I., Cohen, A. O., Barch, D. M., Heitzeg, M. M., . . . Workgroup, A. I. A. (2018). The Adolescent Brain Cognitive Development (ABCD) study: Imaging acquisition across 21 sites. *Dev Cogn Neurosci, 32*, 43-54. doi:10.1016/j.dcn.2018.03.001

Chamberland, M., Raven, E. P., Genc, S., Duffy, K., Descoteaux, M., Parker, G. D., . . . Jones, D. K. (2019). Dimensionality reduction of diffusion MRI measures for improved tractometry of the human brain. *Neuroimage, 200*, 89-100. doi:10.1016/j.neuroimage.2019.06.020

Descoteaux, M., Deriche, R., Knosche, T. R., & Anwander, A. (2009). Deterministic and probabilistic tractography based on complex fibre orientation distributions. *IEEE Trans Med Imaging, 28*(2), 269-286. doi:10.1109/TMI.2008.2004424

Di Tommaso, P., Chatzou, M., Floden, E. W., Barja, P. P., Palumbo, E., & Notredame, C. (2017). Nextflow enables reproducible computational workflows. *Nat Biotechnol, 35*(4), 316-319. doi:10.1038/nbt.3820

Garyfallidis, E., Cote, M. A., Rheault, F., Sidhu, J., Hau, J., Petit, L., . . . Descoteaux, M. (2018). Recognition of white matter bundles using local and global streamline-based registration and clustering. *Neuroimage, 170*, 283-295. doi:10.1016/j.neuroimage.2017.07.015

Gauvin, A. (2016). *Assurance qualité en dissection virtuelle des faisceaux de la matière blanche par tractographie.* Université de Sherbrooke,

Girard, G., Whittingstall, K., Deriche, R., & Descoteaux, M. (2014). Towards quantitative connectivity analysis: reducing tractography biases. *Neuroimage, 98*, 266-278. doi:10.1016/j.neuroimage.2014.04.074

Guberman, G. I., Houde, J. C., Ptito, A., Gagnon, I., & Descoteaux, M. (2020). Structural abnormalities in thalamo-prefrontal tracks revealed by high angular resolution diffusion imaging predict working memory scores in concussed children. *Brain Struct Funct, 225*(1), 441-459. doi:10.1007/s00429-019-02002-8

Guberman, G. I., Stojanovski, S., Nishat, E., Ptito, A., Bzdok, D., Wheeler, A., & Descoteaux, M. (2022). Multi-tract multi-symptom relationships in pediatric concussions. *eLife*. doi:10.1101/2021.04.01.21254814

Kurtzer, G. M., Sochat, V., & Bauer, M. W. (2017). Singularity: Scientific containers for mobility of compute. *PLoS One, 12*(5), e0177459. doi:10.1371/journal.pone.0177459

Rheault, F. (2020). *Analyse et reconstruction de faisceaux de la matière blanche.* Université de Sherbrooke, Retrieved from <https://savoirs.usherbrooke.ca/handle/11143/17255>

Rheault, F., De Benedictis, A., Daducci, A., Maffei, C., Tax, C. M. W., Romascano, D., . . . Descoteaux, M. (2020). Tractostorm: The what, why, and how of tractography dissection reproducibility. *Hum Brain Mapp, 41*(7), 1859-1874. doi:10.1002/hbm.24917

Rheault, F., Poulin, P., Valcourt Caron, A., St-Onge, E., & Descoteaux, M. (2020). Common misconceptions, hidden biases and modern challenges of dMRI tractography. *J Neural Eng, 17*(1), 011001. doi:10.1088/1741-2552/ab6aad

Tournier, J. D., Calamante, F., & Connelly, A. (2007). Robust determination of the fibre orientation distribution in diffusion MRI: non-negativity constrained super-resolved spherical deconvolution. *Neuroimage, 35*(4), 1459-1472. doi:10.1016/j.neuroimage.2007.02.016
